# Antibacterial Activities of *Echinops kebericho* Mesfin Tuber Extracts and Isolation of the Most Active Compound, Dehydrocostus Lactone

**DOI:** 10.3389/fphar.2020.608672

**Published:** 2021-01-25

**Authors:** Serawit Deyno, Andrew G. Mtewa, Derick Hope, Joel Bazira, Eyasu Makonnen, Paul E. Alele

**Affiliations:** ^1^ Department of Pharmacology, Mbarara University of Science and Technology, Mbarara, Uganda; ^2^ Department of Pharmacology, School of Pharmacy, College of Medicine and Health Sciences, Hawassa University, Hawassa, Ethiopia; ^3^ Pharmbiotechnology and Traditional Medicine Center of Excellence (PHARMBIOTRAC), Mbarara University of Science and Technology, Mbarara, Uganda; ^4^ Chemistry Section, Department of Applied Sciences, Malawi Institute of Technology, Malawi University of Science and Technology, Limbe, Malawi; ^5^ MSF Mbarara Research Base, Mbarara University of Science and Technology, Mbarara, Uganda; ^6^ Department of Microbiology, Mbarara University of Science and Technology, Mbarara, Uganda; ^7^ Center for Innovative Drug Development and Therapeutic Trials for Africa, College of Health Sciences, Addis Ababa University, Addis Ababa University, Addis Ababa, Ethiopia; ^8^ Department of Pharmacology and Clinical Pharmacy, College of Health Sciences, Addis Ababa University, Addis Ababa University, Addis Ababa, Ethiopia

**Keywords:** dehydrocostus lactone, essential oil, minimum inhibitory concentration, resazurin assay, optical density, fractions

## Abstract

*Echinops kebericho* Mesfin is traditionally used for the treatment of various infectious diseases. This study investigated antibacterial activity of the essential oil (EO) and the different fractions of ethanol extract. The most active component was isolated and identified. Isolation and purification was accomplished using chromatographic techniques while identification was done by spectroscopic method. Minimum inhibitory concentration (MIC) was determined using the broth micro-dilution method. In bioactive-guided isolation, percent inhibition was determined using optical density (OD) measurement. The MICs of the essential oil ranged from 78.125 μg/ml to 625 μg/ml, and its activity was observed against methicillin-resistant *Staphylococcus aureus* (MRSA, NCTC 12493). Ethyl acetate fraction showed high activity against MRSA (NCTC 12493), MIC = 39.075 μg/ml followed by *Enterococcus faecalis* (ATCC 49532), MIC = 78.125 μg/ml and was least active against *Klebsiella pneumoniae* (ATCC 700603), MIC = 1,250 μg/ml. MIC of hexane fraction ranged from 156.2 µg/ml to *Escherichia coli* (ATCC 49532) to 1,250 μg/ml to *E. coli* (NCTC 11954). The MICs of chloroform fraction ranged from 312.5 to 2500 μg/ml; while butanol fraction could be considered pharmacologically inactive as its MIC value was 2,500 μg/ml for all and no activity against *E. coli* (NCTC 11954). Dehydrocostus lactone was successfully isolated and identified whose MIC was 19.53 μg/ml against MRSA. Dehydrocostus lactone isolated from *E. kebericho* M. showed noteworthy antibacterial activity which lends support to ethnopharmacological use of the plant. Further optimization should be done to improve its antibacterial activities and pharmacokinetic profile.

## Introduction

Antimicrobial resistance (AMR) has resulted in the reemergence of infectious diseases challenges as a contemporary global threat to human health with millions of infections and thousands of annual deaths (Holmstrup and Klausen, 2018). AMR is growing at a faster pace than either discovery of new drugs or alternative approaches to tackling it, leaving common infections untreatable (WHO, 2017). With this resurgence worldwide, the search for novel antimicrobials and alternative approaches to tackling the problem is urgently needed. Modification of existing antibiotics, rediscovering antimicrobials, repurposing antimicrobials, combination treatment, looking for untested sources of antibiotics, and searching for compounds found from chemists around the world are potential sources (Schneider et al., 2017). Plant-based compounds have promising potential as novel antimicrobials or adjuvants in modifying existing antimicrobials (da Silva et al., 2017; Salehi et al., 2019a, Salehi et al., 2019b, Salehi et al., 2020; Yang et al., 2018). A recent review demonstrated a selection of 183 compounds from plants and discussed emerging trends in antibacterial drug discovery from plants (Porras et al., 2020).

Among the many secondary plant metabolites, essential oils (EOs) of different plant sources showed noteworthy antibacterial activities (Dorman and Deans, 2000; Artini et al., 2018) and resistance modulatory effect (Hammer et al., 2012). There is renewed research focused on discovering potential novel antibacterial agents and/adjuvants from plants (da Silva et al., 2017). *Echinops kebericho* Mesfin (Asteraceae) is an endemic medicinal plant used for treatment of various diseases (Alemayehu, 2017). It is used for the management of human and livestock ailments such as wound infections, toothache, tonsillitis, stomachache, gonorrhea, respiratory manifestations, febrile illness, lung *tuberculosis*, trypanosomiasis, typhoid, typhus, common cold, cancer, colic, cough, scabies, malaria, headache, fumigation during childbirth, and as mosquito repellent (Kloos, 1977; Abebe, 1996; Teklehaymanot et al., 2007; Teklehaymanot and Giday, 2007; Abera, 2014; Bitew and Hymete, 2019; Megersa et al., 2019). In veterinary practice, the tubers are powdered and mixed with water and given for black leg disease, respiratory manifestations, liver disease, and skin infections (Yigezu et al., 2014). It is used as a water decoction, infusion, smoke inhaled, orally chewed, and topical sprayed to the affected area (Kloos, 1977; Teklehaymanot et al., 2007; Teklehaymanot and Giday, 2007; Abera, 2014). Its safety was established in animal model (Deyno et al., 2020a) and previous studies revealed the presence of flavonoids, alkaloids, triterpenoids, resins, saponins, and steroids (Abegaz et al., 1991; Toma et al., 2015) and dehydrocostus lactone is also identified as a main component of the EOs (Abegaz et al., 1991; Hymete and Afifi, 1997; Tariku et al., 2011; Ivana, 2015).

The extracts of *E. kebericho* M. exhibited a wide range of pharmacological effects including anti-helminthic, antibacterial, antifungal, antidiarrheal, anti-spasmolytic and antimalarial activities. Methanolic tuber extract and EO showed relatively moderate antimicrobial activities (Belay et al., 2011; Ameya et al., 2016). Antibacterial activities from crude extracts (Desta, 1993; Ashebir and Ashenafi, 1999; Ameya et al., 2016) and EOs (Belay et al., 2011) were reported. Ivan reported activity against *Mycobacterium smegmatis* and significant antimicrobial resistance modulatory effects (Ivana, 2015). Noteworthy activities were also reported against fungi (*Aspergillus flavus* and *Candida albicans*) (Ameya et al., 2016)*,* malaria parasite (*Plasmodium berghe*i) (Toma et al., 2015), Leishmania (Tariku et al., 2011), earthworm (Hymete and Kidane, 1991), Trypanosoma (Abdeta, 2016), EO produced a dose-dependent mosquito repellent activity (Jemberie et al., 2016), and moderate larvicidal activity (Debella et al., 2007).

However, the antibacterial activity against different multi-drug-resistant strains and various solvent fractions have not yet been studied. The spectrum of activity and the most active component of the extract were not elucidated. Herein, we have determined the antibacterial activity of EOs, and fractions of crude extract to eight strains belonging to five species of bacteria. We have isolated the most active compound, identified and determined its activity.

## Methods and Materials

### Extraction of the Plant Material

Fresh plant tuber was collected from Andracha woreda, Sheka zone, Southwest Ethiopia. Details of the collection procedure and identification of specimen and deposited Herbarium were previously described (Deyno et al., 2020a). The tuber was rinsed with running water to remove dirt materials and then air-dried in a shaded place away from Sun light. In four cycles, five hundred grams of the plant material was soaked with 2 L of ethanol for 48 h under agitation. Pounded plant material was dissolved in water in 1/5 (w/v) ratio in a round bottom glass flask and subjected to hydro-distillation for 3 h using the Clevenger type apparatus. The EO was collected, and the volume and weight were measured. The ethanol extract was evaporated on rotary evaporator and dried.

### Separation and Isolation

The dried extract was soaked in distilled water in a separatory funnel where an equal volume of hexane was added, shaken carefully, and allowed to stand until two layers were formed. The hexane layer was separated and the process was repeated three times. Chloroform, ethyl acetate and n-butanol fractions were prepared following the same procedure.

Gradient solvent systems (non-polar to highly polar solvent system) were prepared and tested for best separation of various organic compounds. A column was packed with silica gel that was encountered slowly from the top with mobile phase. When the column was ready, the sample was loaded inside the top of the column. The column was separated from the incoming mobile phase by a thin layer of cotton to prevent the disturbance of the column by an influx of the mobile phase. The mobile solvent was then allowed to flow down through the column.

Weighed dried residue of the ethyl acetate fraction was adsorbed to silica gel (Merck, high-purity grade, pore size 60 Å, 230–400 mesh ASTM) and separated on a column (300 mm height × 22.5 mm diameter). The elution was performed starting from hexane: ethyl acetate (10:0, 10:1, 5:1, 1:1, 1:5, 1:10) followed by mixture of ethyl acetate: methanol (10:0, 10:1, 5:1, 1:1, 1:5, 1:10, 0:10). Fractions collected were analyzed using thin-layer chromatography (TLC) with the help of capillary tube from the lower edge of the TLC plate, and plate is kept in a developing chamber containing suitable solvent system for specific time until the developing solvent reaches the marked edge of the TLC plate. The plate was taken out from the developing chamber, dried and the solvent front was marked by lead pencil. Compound bands/spots visualized on TLC by visual detection, under UV light (254 nm and 360), in iodine chamber and using spray reagent (30% sulfuric acid) for the presence of specific compounds. The visualized spots were marked and the Rf (retention factor) value of each spot was calculated.

### Spectroscopic Analysis

^1^H and ^13^C NMR spectra were recorded on Bruker Avance DMX400 FT-NMR spectrometer using TMS as internal standard, at the Department of Chemistry, Faculty of Science, Addis Ababa University, Ethiopia. Distortionless enhancement by polarization transfer (DEPT) spectra was also acquired by Bruker.

The GC-MS analysis was conducted using Agilent Technologies. The analysis condition was; chromatographic system (7890B GC), detector (5977A MS), and column (DB-5MS, 30 m × 0.25 mm × 0.25 μm). The GC experimental condition was; inlet temperature (260 °C), injection volume (1 μL), split ratio (100). The carrier gas was He with a column flow rate of 1 ml/min. The oven temperature was 40 °C hold for 3 min, then 4 °C/min to 80 °C hold for 7 min, by 4 °C/min to 170 °C hold for 3 min, at 6 °C/min to 230 °C hold for 4 min finally, by 10 °C/min to 270 °C. MS experimental conditions were; ionization mode (EI), EMV mode (gain Factor), gain factor (1), transfer line temperature (280 °C), ion source temp (230 °C), Quad temp (150 °C), solvent delay (3 min), and acquisition mode (scan, 50–550 amu). The compounds were identified by the use of a combination of mass spectrum database search (IMS Terpene Library, NIST MS database), relative retention time (ESO database of EOs) and comparison of mass spectra. Quantitative analysis was performed by peak area normalization (%) measurements (TIC, total ion count) of GC–MS chromatograms.

### Microorganisms

The bacteria strains used were *Staphylococcus aureus* (ATCC 29213), *S. aureus* (MRSA) (NCTC 12493), *Enterococcus faecalis* (ATCC 29212)*, Enterococcus faecalis* (ATCC 49532), *Pseudomonas aeruginosa* (ATCC 27853), *Escherichia coli* (ATCC 25922), *E. coli* (NCTC 11954), and *Klebsiella pneumoniae* (ATCC700603).

### Preparation, Storage and Testing Reagents

Resazurin was prepared in a concentration of 0.015% by dissolving 0.015 g in 100 ml of PBS, vortexed, sterilized by microfiltration and stored at 4 °C (Elshikh et al., 2016).

### Preparation and Serial Dilution of the Extract Solution

Essential oils and fractions/extracts were dissolved in dimethyl sulfoxide (DMSO) for antibacterial activity assay. The final concentration of DMSO was less than or equal to 2%. The stock solution of the extract prepared was 5 mg/ml and was serially diluted with broth. A stock concentration of 128 μg/ml of ciprofloxacin was also prepared and serially diluted.

### Preparation and Standardization of Inoculum

The inocula were prepared following the Clinical and Laboratory Standards Institute (CLSI) recommendation and standardized to 0.5 McFarland turbidity standards equivalent to 1 × 10^8^ CFU/ml using inoculum densitometers. Then it was further diluted to obtain the desired cell density of 5 × 10^5^ CFU/ml (Patel et al., 2015). Briefly, an equivalent of 0.5 McFarland suspensions (1 × 10^8^ CFU/ml) inoculum was prepared and then diluted 1:20 to yield 5 × 10^6^ CFU/ml using saline media. Then, 0.02 ml of this suspension was inoculated into 0.18 ml of broth yielding a final concentration of bacteria approximately equal to 5 × 10^5^ CFU/ml.

### Minimum Inhibitory Concentration Determination

Minimum inhibitory concentration (MIC) was determined using the broth micro-dilution method (Eloff, 1998). CLSI guideline procedures and safety precautions were strictly followed (CLSI, 2018). The 96-well plates were dispensed first with 100 µL MHB then EO, the fractions and isolated compound of 100 µL were added in the first well and serially diluted up to the 10th well. The excess 100 µL was removed from the 10th well. Finally, 100 µL of MHB mixed with inoculum was added from the first to the 11th well. The 11th well contains the broth without the intervention, sterility control, while the 12^th^ well contains broth without inoculum and intervention, fertility control. The final volume in each well was 200 µL.

The bacteria were incubated for 24 h at 35–37 °C. The MIC of DMSO was also evaluated as negative control while ciprofloxacin was a positive control. Prior to use, the broth was checked for sterility and fertility. Microbial growth was indicated by an irreversible change in color from the blue of resazurin to pink resofurin after 20 h of incubation. Minimum bactericidal concentration (MBC) was determined by re-culturing from four well plates which have concentration equal to or greater than the MIC. The plates on which bacterial growth started to be seen were considered MBC. Measurement of optical density (OD) at 620 nm were taken at 0, 2, 4, 18 and 24 h. Graph of time vs. absorbance (OD) vs. concentration was plotted at time 0, 2, 4, 18, and 20 h. The plot at 18 h and 20 h were compared with resazurin assay value for MIC.

### Bioactivity-Guided Isolation

Collected fractions from column chromatography were regrouped based on the TLC plate’s profile reducing the fractions from 67 to 20. These twenty fractions were dried and dissolved in DMSO and antibacterial activity was determined at 0.25 mg/ml concentration to identify the most active fraction. Percent inhibition was calculated using the formula given below for a comparison of fractions activity.

## Results

### Essential Oil Components and Fractions of Ethanol Extract of *E. kebericho* Tuber Yield

**FIGURE 1:**
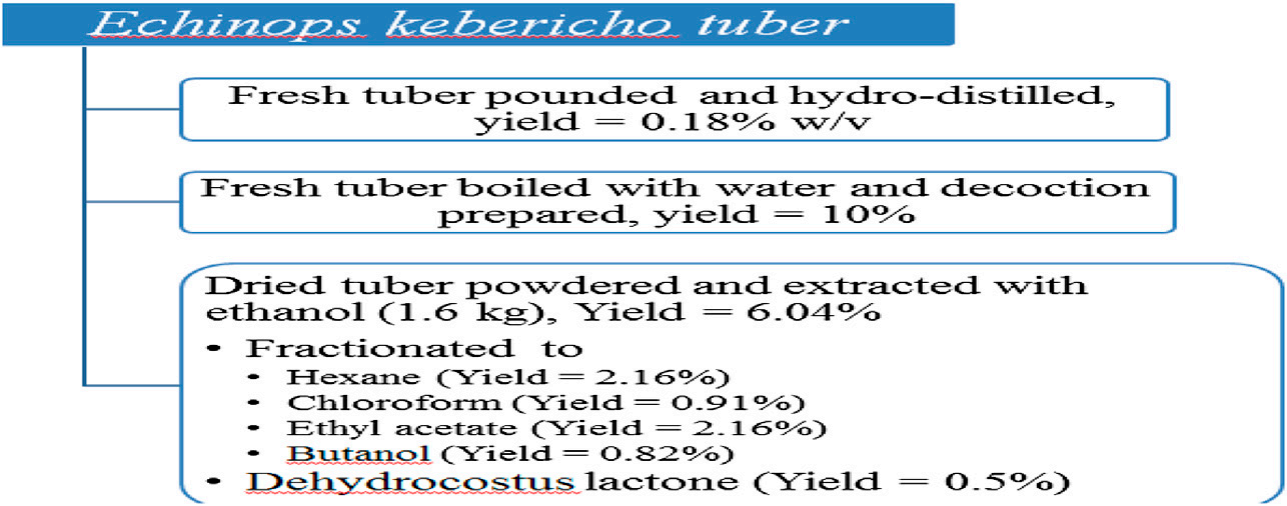
Yields of pounded hydrodistilled and decoction of fresh tuber as well as those of ethanol extract together with its fractions.

The yield of the EO was 0.18% w/v of dried weight. The fractionation of 96.7 g of ethanol extract yielded 34.5 g hexane, 34.6 g ethyl acetate, 14.5 g chloroform, and 13.1 g butanol fractions. The yield of ethanol extract was 6.04%, while that of ethyl acetate residual extract was 2.16%. Finally the yield of the isolated dehydrocostus lactone was 0.5% (Figure 1).

### Phytochemical Screening

The results of preliminary phytochemical screening on ethanol extract as well as its hexane, chloroform, ethyl acetate, and butanol fractions showed presence of different constituents. The major component was terpinoids as summarized in Table 1.

**TABLE 1:** Results of preliminary phytochemical identification tests.

	Extract/fractions test type	Ethanol extract	Hexane fraction	Chloroform fraction	Ethyl acetate fraction	Butanol fraction
1	Ferric chloride test	+	−	−	+	−
2	Test for terpenoids	+	+	+	+	−
3	Dragendorff’s test	−	−	−	−	−
4	Shinoda (flavonoids)	−	−	−	−	−
5	Test of saponins	−	−	−	−	−
6	Glycosides (Benedict’s test)	+	−	−	−	+

### Components of Essential Oil

Hydrodistillation of the tubers of *E. kebericho* M. yielded light yellowish oil (0.18% v/w). Forty-two different EOs were detected; their molecular formula, retention time, and percentage distributions (peak area %) are presented in Table 2. The isoshyobunone was the most abundant compound (12.64%) detected followed by modephene (10.41%), isocomene (8.42%0, β-phellandrene (7.00%), α-pinene (6.96%), DCL (6.52%), β-pinene (6.29%), and β-isocomene (6.08%).

**TABLE 2:** Chemical Composition [% peak area] of EO of *E. kebericho* obtained by hydrodistillation and identified by GC/MS analysis.

No	Name	Formula	Rt	% Area
1	Bicyclo (3.1.0)hex-2-ene 2-methyl-5-(1-methylethyl)	C10H16	10.19	0.13
2	α-pinene	C10H16	10.49	6.96
3	Camphene	C10H16	11.08	0.26
4	Bicyclo (3.1.0)hexane 4 methylene-1-(1-methyethyl)	C10H16	12.04	1.88
5	β-pinene	C10H16	12.24	6.29
6	β-myrcene	C10H16	12.73	0.37
7	α-phellandrene	C10H16	13.36	0.44
8	α-cymene	C10H16	14.20	0.31
9	β-phellandrene	C10H16	14.51	7.00
10	*trans*-β-ocimene	C10H16	15.30	0.34
11	*trans*-verbenol	C10H16O	22.17	1.08
12	Endo-borneol	C10H18O	23.78	0.42
13	Terpinen-4-ol	C10H18O	24.27	0.19
14	(-)-Myrtenol	C10H16O	25.21	0.13
15	Bornyl acetate	C12H20O2	29.48	1.85
16	Silphiperfol-5ene	C15H24	31.09	0.83
17	α-guaiene	C15H24	31.94	3.29
18	7-Epi-silphiperfol-5ene	C15H24	33.06	0.13
19	Modephene	C15H24	33.39	10.41
20	Isocomene	C15H24	33.63	8.42
21	β-elemen	C15H24	33.72	2.46
22	Methyleugenol	C11H14O2	34.22	1.36
23	(A±)-β-isocomene	C15H24	34.45	6.08
24	Caryophyllene	C15H24	34.76	3.13
25	Humulene	C15H24	36.01	1.50
26	Aromandendrene	C15H24	36.16	1.17
27	*trans*-beta-ionone	C13HH20O	36.88	0.39
28	Bicyclogermacren	C15H24	37.37	0.40
29	Cubebol	C15H260	37.50	0.68
30	γ-cadinene	C15H24	37.99	0.41
31	Isoshyobunone	C15H24O	38.15	12.64
32	Germacrene-D-4-ol	C15H26O	40.09	5.25
33	Caryophyllene oxide	C15H24O	40.19	2.45
34	Cyclopenta(c) pentalen-3 (3aH)-one, octahydro-1,2,3a,6tetramethyl	C15H24O	40.64	0.67
35	α-cadinol	C15H26O	42.40	0.71
36	Aromadendrene oxide-(1)	C15H24O	42.51	1.24
37	Pentadecanal	C15H30O	44.22	0.23
38	Costol	C15H24O	45.61	0.50
39	Dihydrodehydrocostus lactone	C15H20O2	50.75	0.42
40	Dehydrocostuslactone	C15H18O2	52.04	6.52
41	Heptacosane	C27H56	56.67	0.57
42	Octacosane	C28H58	62.06	0.49

### Column Chromatography Isolation and TLC Fingerprints

Ethyl acetate fraction of 30 g chromatographed on column gave sixty-seven fractions which were analyzed on TLC plates and pooled based on their fingerprints. Based on their TLC profile, the 67 column fractions were regrouped in 20 fractions. Physical appearance of the fractions F1-5 were oily, F6-8 creamy, F9-14 adhesive mucilage and F15–20 were dry solid.

**FIGURE 2:**
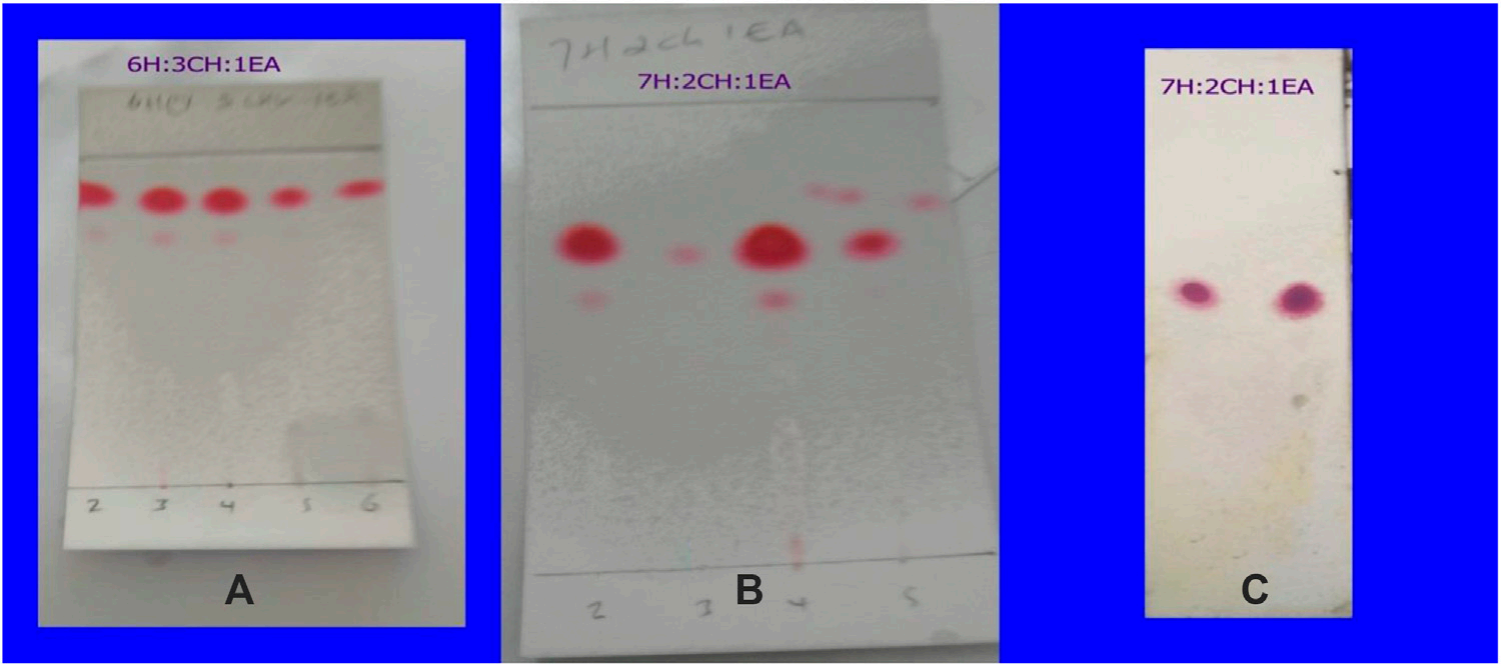
The TLC fingerprint of the first six fractions collected from the column chromatography in different solvent system **(A,B)** and the isolated compound from fractions 1–8 by decantation after crystallization **(C)**.

The TLC profiles of the first four fractions collected were presented in Figure 2. Hexane: chloroform: ethyl acetate (6:3:1) solvent mixture did not provide effective separation while hexane: chloroform: ethyl acetate (7:2:1) did the best separation. The fractions were stored in refrigerator at 2 °C in a refrigerator, and the following day crystal was formed in fractions 1–8. These fractions were separated from the remaining mixture by careful decantation and dried separately after washing gently with hexane for the purification. TLC profiles for each were examined, and those with the same profile were combined. The isolated compound was a white crystal with a melting point of 59–62 °C and dissolved in ethanol, hexane, and acetone but poorly dissolved in water. Its retention time was 20 min in hexane: chloroform: ethyl acetate (7:2:1) solvent system which gave a retention index of 0.614 (Figure 2). The yield of the crystal isolated was 0.5%.

**FIGURE 3:**
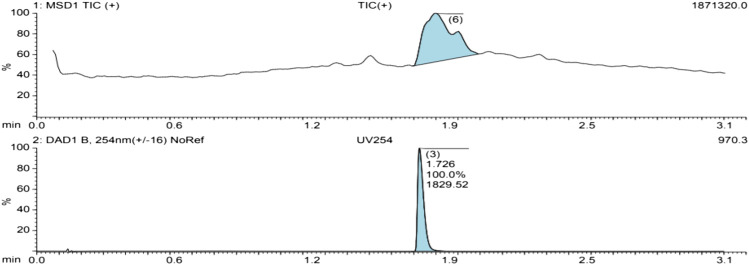
The Total ionic count and UV spectra of an isolated compound.

The UV spectrum of the isolated compound shows a clean single peak at 100% abundance, signifying the purity (Figure 3).

**FIGURE 4:**
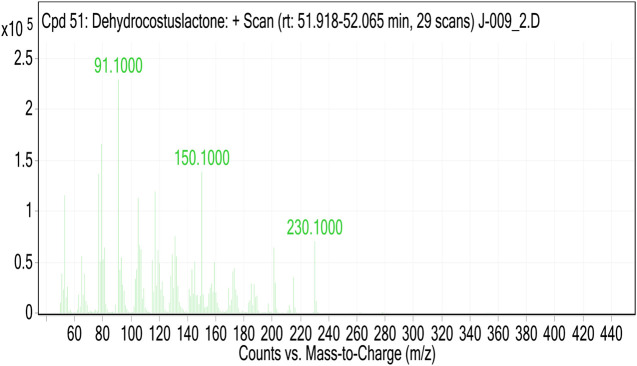
The mass-spectrum of an isolated compound.

The MS of the isolated compound gave [M]+ = 230.1. Analysis of GC-MS and search in libraries identified the compound to be dehydrocostus lactone (Figure 4).

**FIGURE 5:**
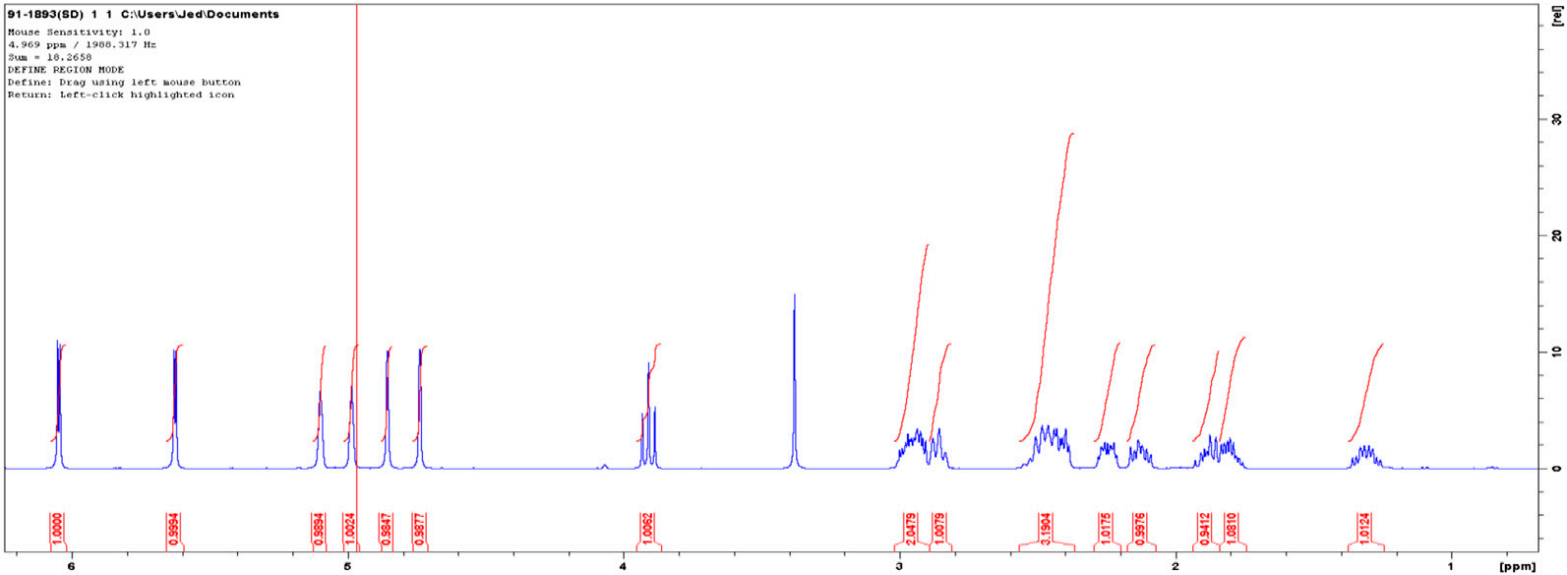
The HNMR spectra of an isolated compound.

HNMR spectra of the isolated compound was; HNMR (DMSO-d, 500 MHz): 1.35 (1H, m), 1.77–1.94 (2H, m), 2.15 (1H, m), 2.26 (1H, m), 2.42 (2H, m), 2.47 (1H, m), 2.88 (1H, m), 2.97 (2H, m), 3.95 (1H, t), 4.76 (1H, s), 4.88 (1H, s), 4.99 (1H, s), 5.10 (1H, s), 5.64 (1H, d), 6.04 (1H, d). Solvent peak was at 2.5 and water residues due to the solvent at 3.33 (Figure 5).

**FIGURE 6:**
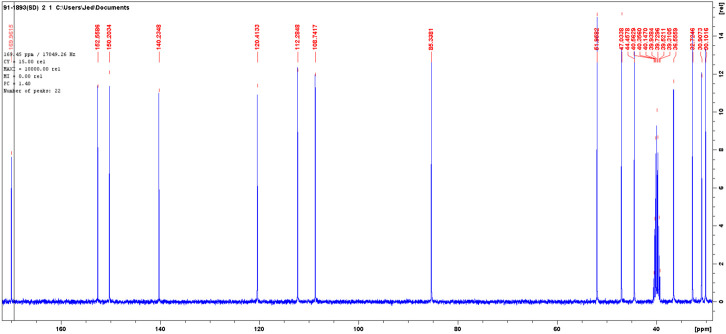
The ^13^C NMR spectra of isolated compound.

The 13C NMR spectra of the isolated compound was (DMSO-*d*, 400 MHz): d30.1 t, 30.9 t, 32.7 t, 36.2 t, 44.5, 47.1, 51.9 d, 85.3 days, 108.7 t, 112.3 t, 120.4 t, 140.2 s, 150.2 s, 152.5 s, 170.0 s. The solvent peak was at 39.5 (Figure 6).

**FIGURE 7:**
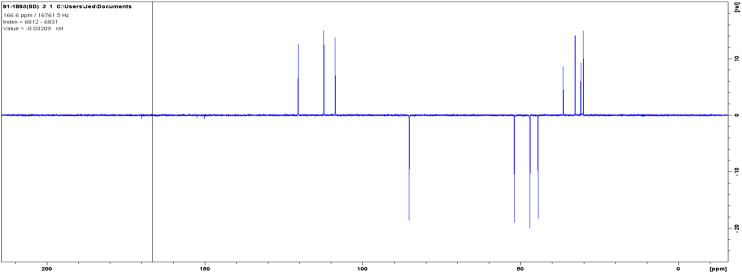
The DEPT NMR spectra of an isolated compound.

The DEPT in comparison with ^13^C NMR (Figure 6) showed the molecule comprises seven methylenes, four methines, and four nonprotonated carbons (Figure 7).

**FIGURE 8:**
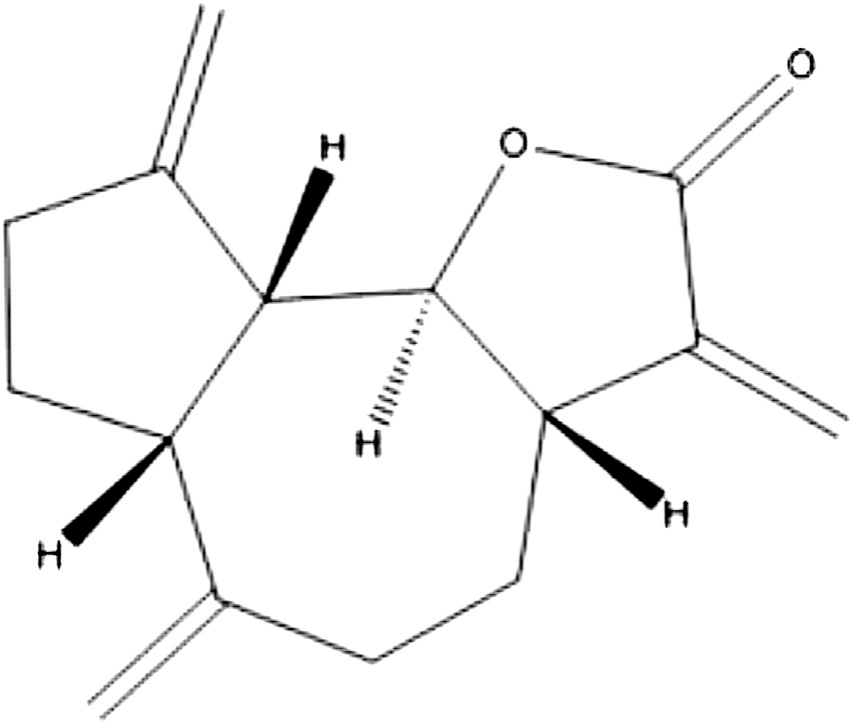
The structural formula of dehydrocostus lactone.

The spectroscopic data given above and the melting point (61–62 °C) confirm that the compound dehydrocostus lactone was successfully isolated and purified which matched with literature (Taniguchi et al., 1995; Lee et al., 2014). The structural formula of the isolated compound was given in Figure 8.

### Effect of Essential Oil of *E. kebericho* M. Tuber on the Bacterial Strains

Moderate antibacterial activity of the EO was observed against the tested strains. The activity against methicillin resistant *S. aureus* was the highest; while least antibacterial activity was observed against *S. aureus* (ATCC 29213), *E. fecalis* (ATCC 49532), *K. pneumonia* (ATCC 700603), *P. aeuroginosa*, ATCC 27853, and *E. coli* (ATCC 25922). The MIC value of the EO ranged from 78.125 to 625 μg/ml. The MBC value ranged from 0.1562 to 2.5 mg/ml. Sensitivity to EO varied even among strains of the same species. DMSO did not show inhibition at concentration used for dissolving (2%) and its MIC value ranged 12.5–25% w/v (Table 3).

**TABLE 3:** Mean Inhibitory and bactericidal concentration of *E.kebericho* EO against bacteria.

	Microorganisms	Essential oil		Ciprofloxacillin	DMSO
		MIC (µg/ml)	MBC (µg/ml)	MIC (µg/ml)	MIC (% W/V)
1	*S. aureus*, ATCC29213	625	1,250	0.5	12.5
2	*S. aureus* (MRSA), NCTC 12493	78.125	156.2	4	12.5
3	*E. fecalis*, ATCC 29212	156.2	156.2	2	12.5
4	*E. fecalis*, ATCC 49532	625	1,250	4	12.5
5	*K. pneumonia*, ATCC 700603	625	2,500	8	25
6	*E. coli*, ATCC 25922	625	1,250	4	25
7	*E. coli*, NCTC 11954	312.5	625	8	25
8	*P. euroginosa*, ATCC 27853	625	1,250	1	12.5

### Effect of Fractions of Ethanol Extract of *E. kebericho Tuber* on the Bacterial Strains

Of all fractions tested, ethyl acetate fraction showed highest activity followed by hexane fraction, and the butanol fraction showed the least activity of all. MRSA was the most susceptible followed by *E. fecalis* (ATCC 49532); while *E. coli* (NCTC 11954) was the most resistant strains among the tested strains. No bactericidal activity was observed against *E. coli* (NCTC 11954) in the tested concentration range (Table 4).

**TABLE 4:** Mean Inhibitory and bactericidal concentrations of *Echinops kebericho* fractions against bacterial species.

	Microorganisms	Hexane fraction		Ethyl acetate fraction		Chloroform fraction		Butanol fraction	
		MIC (µg/ml)	MBC (µg/ml)	MIC (µg/ml)	MBC (µg/ml)	MIC (µg/ml)	MBC (µg/ml)	MIC (µg/ml)	MBC (µg/ml)
1	*S. aureus*, ATCC29213	625	1,250	312.5	—	2,500	—	2,500	—
2	*S. aureus*, NCTC 12493	625	625	039.075	78.125	312.5	625	2,500	—
3	*E. fecalis*, ATCC 29212	625	1,250	156.2	312.5	2,500	—	2,500	—
4	*E. fecalis*, ATCC 49532	156.2	625	78.125	156.2	1,250	—	2,500	—
5	*K. pneumonia*, ATCC 700603	625	1,250	1,250	—	2,500	—	2,500	—
6	*E. coli*, ATCC 25922	312.5	625	312.5	625	1,250	—	2,500	—
7	*E. coli*, NCTC 11954	1,250	—	625	—	2,500	—	—	—
8	*P. euroginosa*, ATCC 27853	312.5	1,250	312.5	625	2,500	—	2,500	—

MIC, minimum inhibitory concentration; MBC, minimum bactericidal concentration.

### Bioactivity-Guided Isolation and Antibacterial Activity of Isolated Compound

**FIGURE 9:**
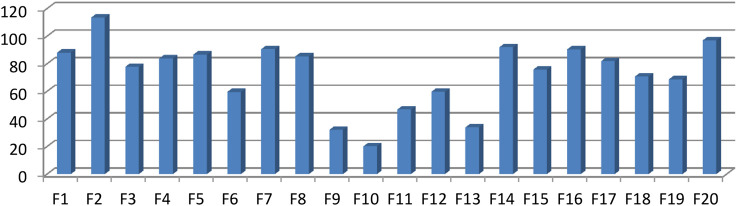
Percent growth inhibition of MRSA strains treated with different column chromatography fractions.

The bioactivity guided isolation showed fraction two (F-2) as the most active component of the twenty fractions as determined using optical density measurement (OD) measurement. Figure 9 depicted percent growth inhibition of each fraction as determined by OD measurement.

**FIGURE 10:**
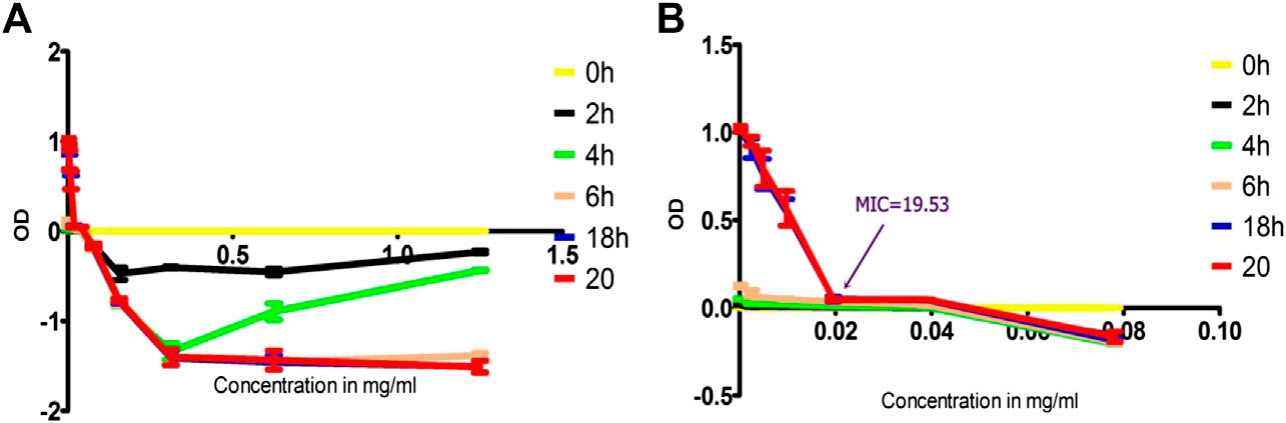
Time-kill analysis using OD values of treated MRSA with isolated compound as function of concentration, **(A)** throughout the range of concentration treated (1.25 to 0.002441 mg/ml) and **(B)** concentration range truncated to values of 0.078125 to 0.002441) to show MIC values clearly.

Fraction two was most active component with MIC = 19.53 μg/ml other fractions were less active and is not single component as illustrated with TLC profile. The Figure 10 depicted the OD difference vs. concentration of the isolated compound at different time of incubation. The OD difference was taken between time 0 h and specific times of measurement. This difference sometimes became negative as result of drop in OD value of the extract solution. Stock solution prepared in DMSO formed a cloudy solution in the beginning but disappeared with increasing temperature and longer stays resulting in dropping of OD values.

**FIGURE 11:**
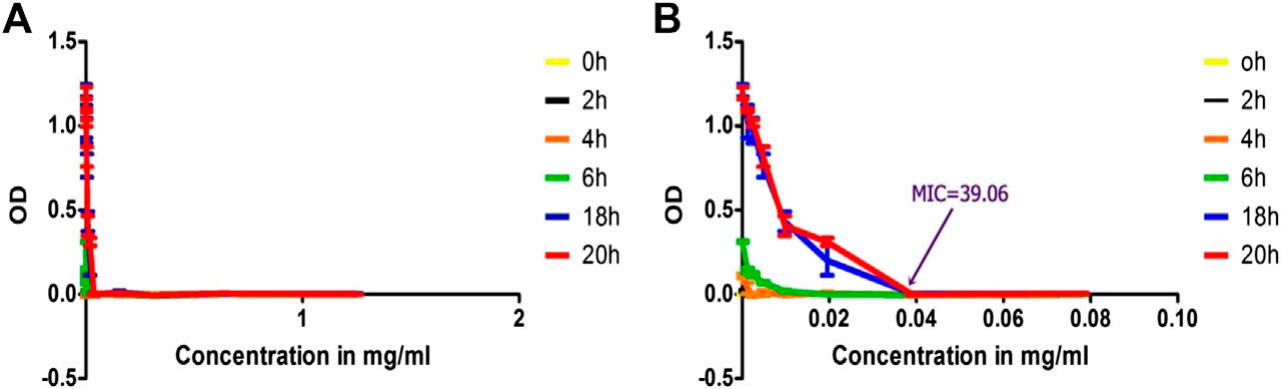
Time-kill analysis using OD values of treated MRSA with benzyl penicillin as function of concentration, **(A)** throughout the range of concentration treated (1.25 to 0.002441 mg/ml) and **(B)** concentration range truncated to values of 0.078125 to 0.002441) to indicate MIC values clearly.

Unlike the OD measurement value of the isolated solution the conventional antibiotics graph did not show significant negative values. This is because of clear solution of the antibiotics in sterile water compared to disappearing cloudy solution of the isolated compound (Figure 11).

## Discussion

Phytochemical screening revealed terpenoids as major components in *E. kebericho* extract. This is consistent with previous profiling of the EO components (Hymete et al., 2007; Tariku et al., 2011) where the Eos majorly contain terpenoids. We successfully isolated and identified dehydrocostus lactone whose spectroscopic data matched well with literatures (Taniguchi et al., 1995; Lee et al., 2014). It was the first of its kind to isolate dehydrocostus lactone from *E. kebericho* M. and determine its antibacterial activity though it was previously isolated from other plants (Chhabra et al., 1998; Li et al., 2005; Lee et al., 2014). Dehydrocostus lactone was previously detected from EO (Tariku et al., 2011) and in the smoke of the plant (Turek et al., 2016). The yield of dehydrocostus lactone (0.5%) was greater than that of the EO (0.18%). This could be due effectiveness of solvent extraction method compared to hydrodistillation. Hydrodistillation could damage the components and reduce the yield. This yield was less than that of Saussurea root (1.3%) (Okugawa et al., 1996) and artemisinin (1%) from *A. annuais*, which is also a sesquiterpene (Van Der Kooy and Verpoorte, 2011).

Eight strains of bacteria belonging to five species were investigated for antimicrobial susceptibility to EO and fractions of *E. kebericho* M. demonstrated a range of susceptibilities. Essential oil and ethyl acetate fraction exhibited high activity against most bacteria tested compared to other fractions. The highest antibacterial effect of ethyl acetate fraction could be attributed to the most active chemical compound/s present in the fraction. The results of previous studies (Kotzé and Eloff, 2002) were in agreement with those of the present study where extraction by different solvents showed different activities. The highest antibacterial activity of ethyl acetate fraction compared to crude extract and/or other fractions were also reported in previous studies (Gnan and Demello, 1999; Geidam et al., 2007).

Significant variation in the activity of the same fraction against strains of the same species was observed in the case of *S. aureus* and *E. feacalis*. In both cases, resistant strains were more susceptible compared to wild type. MRSA, mecA positive, was more susceptible to EO and ethyl acetate fraction compared to a wild type strain ATCC 29213. Similar finding was also noted with epigallocatechin Gallate b (plant extract) where *S. aureus* (ATCC 29213) was less susceptible compared to MRSA strains, possibly due to an increasing affinity of the extract to mutated proteins of MRSA (Betts et al., 2015). Mutation is an energy demanding, and results in increased susceptibility to a naive stress compared to a wild type. Previous studies had outlined increased overall antibiotic susceptibility to new antimicrobial agents in *S. aureus mutants* (Ling and Berger-Bächi, 1998). The increased susceptibility of mutant strains of *S. aureus* and *E. feacalis* in the current study could indicate enhanced susceptibility of mutants to a naive stress.

Gram-positive species were more susceptible to both the EO and fractions compared to Gram-negative species. Greater susceptibility of Gram-positive species compared to Gram-negative was ascribed to differences in their cell wall structures. The Gram-negative outer membranes are diffusional barriers making bacteria less susceptible to many antimicrobial agents (Ghai and Ghai, 2017). Gram-positive mutant strains were susceptible compared to wild type but more resistance was observed in Gram-negative mutant strains. This could be due to membrane alteration in gram-negatives favoring more resistance which was also shown in previous study where reduced outer membrane permeability contributed to enhanced resistance of *E. coli* O157:H7 to antimicrobial agents (Martinez et al., 2001). Gram-positive bacteria are known to be more susceptible to EO than Gram-negative bacteria. *E. coli* was least susceptible to EO in the present study which was consistent with the previous finding (Inouye et al., 2001). Resistance of Gram-negative bacteria was in most cases ascribed to the presence of complex resistant outer membrane (Onawunmi and Ogunlana, 1985; Mann et al., 2000).

The OD measurement worked well for MIC determination, time-kill analysis and bioactivity guided isolation. However, the concentration-OD curves at various time showed negative values in the case of isolated compound and the EO. This might mostly likely be due to turbidity change after incubation with increasing temperature. Higher concentrations of the isolated compound and the EO mixture with DMSO and water at 21 C were more turbid and with more OD values. But after incubation at 35–37 C, the mixture gradually became clear solution which could be due to increased solubility as the temperature increased. This reduces the OD measurement from baseline values resulting in negative OD difference. This mix-up was avoided by truncating the graph around the MIC value. When truncated, the graph indicated complete growth cessation and MIC value clearly. These negative values were not observed with a standard antibiotic (penicillin-G) as they form clear solution without turbidity.

Dehydrocostus lactone, the isolated compound, was two times more potent than penicillin-G. This is the first report on the antibacterial compounds isolated from *E. kebericho* M. against MRSA*.* The potency of isolated compound was two times that of ethyl acetate fraction and four times that of the EO of the same plant. This could be due to the presence of numerous constituents that reduced the amount of the active isolated components in the mixture or increased amount of the isolated compound due to removal of the impurities. The amount of DCL in the ethylacetate fraction was 26.66%. The remaining component (74.34%) could be expected to have lesser antibacterial activity compared to the isolated compound.

Ameya and his colleagues determined the antibacterial and antifungal activities of *E. kebericho* crude extract. The extract demonstrated noteworthy activities against *S. aureus*, *E. coli* and *E. faecalis* (Ameya et al., 2016)*.* The current study, however, went further and isolated the most active compound (DCL), identified and determined its antibacterial activities. In this regard, the current study could be considered the continuation of the previous study (Ameya et al., 2016). Wild type and resistant strains of the same species compared; surprisingly, the resistant (MRSA) was found to be more susceptible to the plant extract compared to the wild one.

Dehydrocostus lactone isolated from other plants showed noteworthy activities against; antibiotic-susceptible and resistant *Helicobacter pylori* strains (MIC = 4.0–6.7 μg/ml) (Lee et al., 2014), *Mycobacterium tuberculosis* H37Rv (12.5 μg/ml) (Luna-Herrera et al., 2007), *M. tuberculosis* (2 μg/ml), and *Mycobacterium avium* (16 μg/ml) (Cantrell et al., 1998). These activities ranges are almost similar to current findings and the little differences could be attributed to interspecies variation. Its activity decreased with the oxidized products perhaps due to decreased lipophilicity of guaianolide required for enhanced anti-mycobacterial activity indicating that the antibacterial activity is most likely associated to transport of guaianolide through the outer lipid layer (Cantrell et al., 1998). The antibacterial activity of highly non-polar compound could be attributed to their membrane disruptive property (Vasconcelos et al., 2018), damage to cell membrane; alter lipid profile; inhibit ATPases, cell division, membrane porins, motility, and biofilm formation; and anti-quorum sensing effects (Zhang et al., 2016). The findings of the current study where more non-polar fractions, EO and highly lipophilic DCL possess higher antibacterial activities could possibly indicate membrane disruptive potential.

The antibacterial activities of sesquiterpenes were mostly related to guaianolide lactone and cyclopentenone moiety (Gibbons, 2004). The guaianolide in sesquiterpene lactone showed remarkable activity against methicillin sensitive *S. aureus* (MSSA) (MIC 38 μg/ml) and the activities were suggested to depend on the presence of a beta unsubstituted cyclopentenone ring moiety (Kuo-Hsiung et al., 1977). A guaianolide from *Artemisia gilvescens* showed excellent activities against MRSA (MIC = 1.95 μg/ml) (Kawazoe et al., 2003). Sesquiterpene curzerenone from myrrh along with other sesquiterpene mixtures showed excellent anti-staphylococcal activity (MIC = 0.7 μg/ml) (Kawazoe et al., 2003). Any modification on DCL for better activity should be made on these moieties.

The previous (Cantrell et al., 1998; Luna-Herrera et al., 2007) and the current studies showed worthy and promising antibacterial activity of DCL. However, it requires optimization to get ideal antibacterial drug hit as this has to possess higher molecular weight and increased polarity (O’Shea and Moser, 2008). Drugs with activity against only Gram-positive bacteria have much less restriction in molecular weight, especially if the target is located in the peptidoglycan matrix or on the outer surface of the underlying lipid bilayer as permeation through the inner lipid membrane is not required for the activity (O’Shea and Moser, 2008). As more activity was observed against Gram-positive compared to Gram-negative bacteria; the compound could be expected to have much less restriction in molecular weight. On the other hand, increasing the polarity of DCL reduced the activity as the activity is most likely depends on the transport through the outer lipid layer of the membrane (Cantrell et al., 1998).

Appropriate pharmacokinetic profile is crucial for improved effectiveness of a particular drug. Oral absorption of DCL was established to be good (JingZe et al., 2014; Zhang et al., 2015) but much larger peak concentration and the total amount of DCL in the body was observed with intravenous than oral administration (Peng et al., 2014). Intravenous administration might be a better route for clinical use and the compound lacks ideal antibacterial property as it shows very poor solubility in water and requires optimization for better activity. As the oral route remains the main route for the administration of herbal medicine, the bioavailability of DCL affects therapeutic effectiveness. The lead optimization for better pharmacodynamics and pharmacokinetic profile is required to be investigated. Furthermore, the safety profile and mechanism of antibacterial action of DCL should be investigated.

## Data Availability Statement

The original contributions presented in the study are included in the article/Supplementary Material, further inquiries can be directed to the corresponding author.

## Ethics Statement

The animal study was reviewed and approved by Faculty Research Committee and approved by the Research Ethics Committee (REC) of Mbarara University of Science and Technology, Uganda. It was also registered with Uganda National Council for Science and Technology (UNCST) with reference number HS398ES.

## Author Contributions

SD conceived the research idea. SD and DH conducted antimicrobial activity study. SD and AM conducted spectroscopic analysis for isolated compound. EM, JB, and PA developed the concept, monitored and mentored the proposal development. SD wrote the draft manuscript. All authors revised, edited and approved the final manuscript.

## Funding

This study was funded by World Bank through Pharm-Biotechnology and Traditional Medicine Center (PHARMBIOTRAC), African Center of Excellence II (ACE-II) Project. The funder contributed research money and stipends (SD) but was not involved in planning and implementation of the study.

## Conflict of Interest

The authors declare that the research was conducted in the absence of any commercial or financial relationships that could be construed as a potential conflict of interest.

## Abbreviations

ATCC, american type culture collection; CLSI, clinical and laboratory standards institute; DCL, Dehydrocostus lactone; DMSO, Dimethyl sulfoxide; EO, Essential oil; MBC, minimum bactericidal concentration; MHB, Muller-Hinton broth; MIC, minimum inhibitory concentration; MRSA, Methicillin-resistant *Staphylococcus aureus*; NCTC: national collection of type cultures; OD, optical density.
